# Post-hospital medical respite care for homeless people in Denmark: a randomized controlled trial and cost-utility analysis

**DOI:** 10.1186/s12913-020-05358-4

**Published:** 2020-06-05

**Authors:** Camilla Bring, Marie Kruse, Mikkel Z. Ankarfeldt, Nina Brünés, Maja Pedersen, Janne Petersen, Ove Andersen

**Affiliations:** 1 Clinical Research Centre, Copenhagen University Hospital Amager and Hvidovre, Kettegaard Alle 30, 2650 Hvidovre, Denmark; 2 Danish Centre for Health Economics, University of Southern Denmark, Odense, Denmark; 3 Center for Clinical Research and Prevention, Bispebjerg and Frederiksberg hospital, Frederiksberg, Denmark; 4 Patient Care, Copenhagen University Hospital Amager and Hvidovre, Hvidovre, Denmark; 5 Department of Cardiology, Bispebjerg and Frederiksberg Hospital, Frederiksberg, Denmark; 6 Section of Biostatistics, Department of Public Health, University of Copenhagen, Copenhagen, Denmark; 7 Emergency Department, Copenhagen University Hospital Amager and Hvidovre, Hvidovre, Denmark; 8 Institute of Clinical Medicine, University of Copenhagen, Copenhagen, Denmark

**Keywords:** Medical respite care, Homeless, Acute hospitalization, Economic evaluation, Cost-utility analysis, Cost-effectiveness, Randomized controlled trial, RCT

## Abstract

**Background:**

Being homeless entails higher mortality, morbidity, and prevalence of psychiatric diseases. This leads to more frequent and expensive use of health care services. Medical respite care enables an opportunity to recuperate after a hospitalization and has shown a positive effect on readmissions, but little is known about the cost-effectiveness of medical respite care for homeless people discharged from acute hospitalization. Therefore, the aim of the present study was to investigate the cost-effectiveness of a 2-week stay in post-hospital medical respite care.

**Methods:**

A randomized controlled trial and cost-utility analysis, from a societal perspective, was conducted between April 2014 and March 2016. Homeless people aged > 18 years with an acute admission were included from 10 different hospitals in the Capital Region of Denmark. The intervention group (*n* = 53) was offered a 2-week medical respite care stay at a Red Cross facility and the control group (*n* = 43) was discharged without any extra help (usual care), but with the opportunity to seek help in shelters and from street nurses and doctors in the municipalities. The primary outcome was the difference in health care costs 3 months following inclusion in the study. Secondary outcomes were change in health-related quality of life and health care costs 6 months following inclusion in the study. Data were collected through Danish registries, financial management systems in the municipalities and at the Red Cross, and by using the EQ-5D questionnaire.

**Results:**

After 3 and 6 months, the intervention group had €4761 (*p* = 0.10) and €8515 (*p* = 0.04) lower costs than the control group, respectively. Crude costs at 3 months were €8448 and €13,553 for the intervention and control group respectively. The higher costs in the control group were mainly related to acute admissions. Both groups had minor quality-adjusted life year gains.

**Conclusions:**

This is the first randomized controlled trial to investigate the cost-effectiveness of a 2-week medical respite care stay for homeless people after hospitalization. The study showed that the intervention is cost-effective. Furthermore, this study illustrates that it is possible to perform research with satisfying follow-up with a target group that is hard to reach.

**Trial registration:**

ClinicalTrials.gov Identifier: NCT02649595.

## Background

Homelessness is increasing in high-income countries [1–3]. Homeless people generally have higher mortality and morbidity, including a high prevalence of psychiatric disorders and substance use problems [1, 4, 5]. Overall, this leads to substantially higher use of health care services and more frequent hospitalizations, resulting in higher health care costs compared to the general population [6–8]. Over the last decade, the average number of inpatient days has decreased, and there has been a tendency to provide more health care on an outpatient basis [9]. This can be particularly challenging for homeless people, as living on the street after hospitalization is not conducive to recovery [10]. Doran et al. showed that 70.3% of all hospitalizations of homeless people result in either readmission or an emergency department visit within 30 days after discharge, with approximately 75% of the hospital readmissions occurring within the first 2 weeks after hospital discharge [11]. Another consequence of homelessness is prolongation of in-hospital stay due to postponement of discharge [8], also termed “alternative level of care”, referring to patients who no longer need the treatment provided in acute care hospitals [12].

A possible solution could be medical respite care, which is described by the National Health Care for the Homeless Council as ‘acute and post-acute care for persons experiencing homelessness who are too ill or frail to recover from a physical illness or injury on the streets but are not ill enough to be in a hospital’ [13]. Medical respite care has attracted increased attention [14, 15], primarily because it has been shown to reduce future hospital admissions, as well as length of in-hospital stay, among homeless people discharged from an acute hospitalization, thereby releasing beds for people in need of acute medical care [16]. In addition, medical respite care enables cooperation with other social and health care services, such as primary care, social services, and outpatient medical care [14]. Staying in respite care and the improved opportunity to cooperate with municipalities seems to have a positive impact on substance abuse problems [15, 17]. Thus, it seems likely that medical respite care for homeless people can be part of the solution to a complex problem. However, the cost-effectiveness of medical respite care for homeless people needs to be investigated. In this randomized controlled trial, we investigated whether a 2-week medical respite care stay after an acute hospitalization is a cost-effective intervention for homeless people from a societal perspective. Furthermore, we investigated whether the intervention can improve health-related quality of life.

## Methods

The study is a collaboration between Red Cross Copenhagen and Amager and Hvidovre Hospital, a large university hospital in the Danish capital region in the suburbs of Copenhagen. The Red Cross provided the medical respite care facilities, and Amager and Hvidovre Hospital planned and conducted the scientific evaluation and outcome assessment in relation to the present study.

### Study design

The study “Bridge Copenhagen – medical respite care for homeless people” is a pragmatic randomized controlled trial including a health economic evaluation.

### Setting

In Denmark, access to health care services, such as hospitalization and general practitioners, is free of charge, as most of the health care sector is financed by taxes. Hospitals are responsible for treating diseases in the acute phase, as well as the outpatient follow-up. The municipalities are responsible for rehabilitation after hospitalization, as well as other primary health care services, including services aimed at homeless people after discharge from the hospital. In Copenhagen, the health care services for homeless people includes street clinics with doctors and nurses, shelters, rehabilitation centers, drop-in centers, and inpatient and outpatient therapy for use of drugs and alcohol. The services are operated by municipalities and non-governmental organizations (NGOs).

The hospitals in the Capital Region of Denmark have employed nurses with specific training and experience in working with socially marginalized people, including homeless people and people with problematic use of alcohol and drugs [18]. These social nurses assist in optimizing hospital treatment and care for marginalized patients. Upon discharge, they can facilitate contact with shelters and municipalities for further help. At the time of this study, the social nurses were the ones referring patients for stays in medical respite care [18].

### Participants

Acutely admitted patients age ≥ 18 years who were self-reported homeless or functionally homeless and were going to be discharged from one of the 10 hospitals in the Capital Region of Denmark were offered inclusion in the study. The term ‘functionally homeless’ refers to a person who formally has an address but cannot stay there due to, for example, violence, threats, or poor condition of the residence [19]. If participants were not able to be alone during the night, use the stairs to the first floor, were not self-reliant in daily activities, or were illegal immigrants, they were excluded due to internal rules at the center. If the social nurses or the principal investigator was in doubt about whether the patient fulfilled the inclusion criteria, they would consult the head of the medical respite care center. Questionnaires, patient information, and the consent form were available in Danish, English, Polish, Russian, and French; patients who could not read these languages were not included. There were no restrictions regarding drug and alcohol use. Because the study was conducted as a pragmatic randomized study in a normal clinical setting, we were not able to produce a full flowchart of all the homeless patients attending the hospitals during the study period. However, we collected data 1 month in September 2015 about all patients who were in contact with the social nurses but did not comply with inclusion criteria.

### Post-hospital medical respite care intervention

In April 2014, Red Cross Copenhagen opened a medical respite care center with four beds for homeless people discharged after hospitalization. After 6 months, the capacity expanded to eight beds. The place was led by a paid registered nurse (RN) and primarily staffed with volunteers. The medical respite care center offered a 2-week stay including three meals a day, free of charge. The patients were accommodated in double rooms with attached bathrooms. The RN assisted with uncomplicated nursing tasks, such as caring for wounds, helping with medicine, catheter care, and monitoring of blood glucose, and helped patients with social issues, such as housing and communicating with municipalities about the provision of further services. The Red Cross medical respite care center differed from the services in the municipality in two important ways: it was free of charge, and there were no restrictions regarding drug and alcohol use. The medical respite center was financed by the government during the study period.

The control group was discharged from the hospital with help from the social nurses, but independently had to seek help from the described standard municipal facilities, such as shelters, street nurses, and doctors.

### Randomization and blinding

The randomization was conducted in blocks of four with a 2:2 ratio and stratified for each hospital to ensure that each hospital had a chance to refer the homeless patients and that there was broad representation of patients from all over the Capital Region. The randomization was performed when the patient was ready for discharge. Participants were included by one of the 10 social nurses. When a possible participant for the study was identified, the social nurse informed the patient about the medical respite center and the study design. The patient signed an informed consent form and answered the health-related quality of life (EQ-5D-5 L) questionnaire [20]. The social nurse called the medical respite center, where the employees drew a sealed opaque envelope that concealed the group to which the participant was assigned. Thus, the actual draw took place in the respite center, where group assignment was also revealed. Afterwards, the primary investigator double checked the randomization. The principal investigator was the only one who knew the randomization code and prepared the envelopes. The first six patients included in the study were all assigned to the intervention group without randomization to get the medical respite care program up and running. Blinding of the participants was not possible due to the nature of the study. However, data analysis was performed blinded by a single researcher (M.K.).

### Study perspective

This study is one of a few randomized controlled studies performed in acutely admitted homeless patients, a socially stigmatized group [21]. The perspective of the economic evaluation is societal. The preconception is that it is challenging to perform randomized controlled trials with follow-up in a population of homeless people [22]. Therefore, we initially designed the period of the economic evaluation to be 3 months in an attempt to prevent the study from suffering from a high drop-out rate [22]. The societal perspective of the economic evaluation was based on costs from the health and social care sector; however, some costs (e.g., prison) were not included. We included all costs from the Danish health care system, municipalities, and the medical respite care center. The analysis will not include patients’ or families’ use of time because homeless people often have limited or no contact with family and peers and are primarily supported by transfer income [23, 24]. Any costs or savings related to participants finding stable housing as a positive consequence of the medical respite care stay could be relevant to the investigation but is beyond the scope of this study.

### Outcomes and measures

The primary outcome was a difference in health care costs for a period of 3 months after hospital discharge. Data on the costs of the health care services provided in hospitals and at general practitioners were extracted from the National Patient Register and the National Health Insurance Service Register through Statistics Denmark. Costs in the municipalities were collected through databases in the municipalities in the Capital Region of Denmark. We received information about the type of health care service, date of accounting, and costs. The costs of the medical respite care center were retrieved through Red Cross Copenhagen. Costs in Danish kroner (DKK) were converted to Euro (€) using the exchange rate €1 = 7.5 DKK [25]. Data about immigrants without a Danish social security number were retrieved manually by their provisory social security number in the medical journals.

Secondary outcomes were a change in health-related quality of life (HRQoL), health care costs after a period of 6 months, and health care costs grouped into elective health care costs, acute health care costs, and social costs after a period of 6 months. The HRQoL were measured by EQ-5D-5 L at baseline, after 2 weeks, and after 3 months. The measurement at 3 months was decided after inclusion of the first 25 participants because the 2-week time horizon was deemed rather short and we found it was possible to perform a satisfying follow-up after 3 months. The EQ-5D-5 L is a generic and validated questionnaire that measures HRQoL in five dimensions: mobility, self-care, usual activities, pain/discomfort, and anxiety/depression [26]. Each question has five possible answers: no problems, slight problems, moderate problems, severe problems, or unable to/extremely. The answers can give a total of 3125 possible health states that can each be converted to an index value using the cross-walk value set [26]. The index values are used to calculate quality-adjusted life years (QALYs), which is the outcome presented in this study. Compared to the EQ-5D-3 L, the EQ-5D-5 L is more sensitive and able to detect smaller differences in interventions and treatments [20]. The QALY measurement had the same time horizon as the cost measurements: 3, 6, and 12 months.

Follow-up was conducted mainly by the primary investigator either as personal interviews face-to-face or by phone. Nurses and social workers in the municipalities assisted in collecting the follow-up questionnaires based on instructions from the primary investigator.

Participant characteristics and covariates were collected from Danish registries: age, gender, having a social security number, source of income, highest completed education, admissions 2 years prior to inclusion, psychiatric diagnoses, and the Charlson Comorbidity Index, indicating the burden of diseases [27]. Use of drugs and alcohol and housing status were collected from a database registering services provided by the social nurses, and information about what kind of help the intervention group received at the medical respite center was retrieved from the database of the medical respite center.

Sample size was calculated to detect a 25% difference in cost between the two groups. With estimated health care costs of €13,402 in the control group, standard deviation of €5259, a significance level of 5%, and a power of 80%, 96 patients were needed assuming 20% loss to follow-up.

### Assessment of costs

To investigate whether a medical respite care stay influenced the participants’ use of social and health care services, we split the costs into three categories: elective health care costs, acute health care costs, and social costs. Elective health care costs relate to all planned health care services, such as visits to the general practitioner, outpatient visits, elective hospitalization, rehabilitation in the municipalities, and inpatient and outpatient therapy for use of drugs and alcohol. Primary health care tariffs were used for costing primary health care, and a standard outpatient and bed-day tariff was used for costing hospital services. Acute health care costs comprise acute admissions and emergency department visits, as well as in-hospital days after inclusion. The latter is important because some participants remained hospitalized after inclusion, either because their condition deteriorated, or because they waited for an alternative level of care. The social costs include the social and health care services delivered in the municipalities, inter alia, the estimated hours of contact with social workers, and lodging at shelters. Costs related to the medical respite care stay were also categorized as a social cost, but only calculated for the randomized intervention group.

Hospital services and health care use in primary care were evaluated using the National Patient Register and National Health Insurance Service Register through Statistics Denmark [28, 29]. Information on the purchase of prescription drugs filled at pharmacies is found in the Danish National Prescription Register [30]. It is customary for the hospitals to dispense medicine to homeless people at discharge without registering this in the electronic health record. Furthermore, pharmaceuticals are handed out in street clinics, where patients are sometimes treated anonymously. Therefore, we will conduct the analysis with and without data from the Danish National Prescription Register. Information about health care services in the municipalities are retrieved from both financial management systems and manually collected information about delivered services in street clinics. We received information about the type of health care service, date of accounting, and cost of the municipal service.

The municipal costs were valued using the unit costs given in Table 1. Health care professionals’ time was valued based on the 2016 and 2017 mean wages for registered nurses and staff physicians (€33 and €62 per hour, respectively) [33, 34]. Costs from the medical respite care stay were obtained from the financial management system at Red Cross Copenhagen. Costs consisted of salaries for the daily leader and other staff, including a consultant working in the head office at Red Cross Copenhagen, food, washing of clothes and linen, and cleaning and nursing requisites. Time spent by the volunteers was valued by the number of volunteer hours spent at the medical respite care center multiplied by an estimated unit cost of €25, including perks. This estimate is based on the mean salary for an unskilled worker in the welfare sector [31]. Actual days in the medical respite care stay was registered and reported as median (IQR).

**Table 1:** Cost components and unit costs

Type of resource used	Unit	Mean unit cost € (IQR)	Source
***Medical Respite Care***			
**Running expenses**^**a**^	Per day	142	Financial management system at Red Cross Copenhagen
**Nurse (head of medical respite care)**	Per hour	36	Financial management system at Red Cross Copenhagen
**Volunteers**	Per hour	25	FOA (trade union) [31]
**Employees (medical respite care)**	Per hour	36	Financial management system at Red Cross Copenhagen
***Health Care***			
**Inpatient**	Per admission	3222 (68,964)	National Patient Register, DRG-coded [29]
**Outpatient**	Per visit	227 (3398)	National Patient Register, DRG-coded [29]
**Emergency department**	Per visit	77 (508)	National Patient Register, DRG-coded [29]
**Day in psychiatric ward**	Per admission day	467	National Patient Register, DRG-coded [29]
**Hospital days after inclusion**	Per day	467	National Patient Register, DRG-coded [29]
**General practitioner**	Per hour	92	The Danish Medical Association [32]
**Prescription drugs**	Per package	10 (409)	The Danish National Prescription Registry [30]
***Municipal Services***			
**Alcohol and drug therapy (inpatient)**^**b**^	Per month	2400	Financial management system at municipalities
**Alcohol and drug therapy (outpatient)**^**b**^	Per visit	467	Financial management system at municipalities
**Rehabilitation**^**b**^	Per hour	120	Financial management system at municipalities
**Drop-in center for adults with special needs**^**b**^	Per trajectory	347	Financial management system at municipalities Financial management system at municipalities
**Shelters for adults with social problems**^**b**^	Per month	4700	Financial management system at municipalities
**Nurse and care**^**b**^	Per hour	33	The Danish Nurses’ Association [33]
**Personal assistance for adults with special needs**^**b**^	Per treatment	440	Financial management system at municipalities
**Assistive devices for adults with special needs**^**b**^	Per device	186 (135)	Financial management system at municipalities
**Administrative costs**^**b**^	Per service	56 (10)	Financial management system at municipalities
**Personal financial supplement**^**b**^	Per transfer	1525 (1221)	Financial management system at municipalities
**Home care**	Per hour	25	FOA (trade union) [31]
**Street nurse**	Per hour	33	The Danish Nurses’ Association [33]
**Physician**	Per hour	62	The Danish Medical Association [32]

^a^Includes food, cleaning, nursing articles, laundry, and perks for volunteers

^b^Representative price from the municipalities

Costs in the primary analyses included all costs incurred at hospitals, general practitioners, and medical specialists, as well as the costs of prescription drugs and costs related to services delivered in the municipalities and the medical respite care center. Income transfers were excluded. Data from municipalities were double-checked in an audit by the principal investigator and M.K. Data from municipalities contained information on the date of accounting, but not the date of delivery of service; therefore, the analysis of municipal data was only performed for the 6-month period.

### Statistical analysis and cost-utility analysis

Demographic information and information on transfer incomes and health history are presented as means, standard deviations, and percentages. The cost-utility analysis was performed as an intention-to-treat stochastic cost-effectiveness analysis because both costs and effects were determined using data from the participants in the study [35]. The primary analysis compared costs during the 3 months following inclusion in the study for both groups and was adjusted for costs for the 3 months preceding inclusion.

For the secondary analysis with a 6-month follow-up, we adjusted for cost 6 months prior to inclusion. Post-hoc, we decided to also conduct the analyses for a 12-month period. The baseline covered a period of 12 months, equivalent to the period following inclusion in the study. Therefore, the cost regression was conducted as a difference in difference analysis as shown in model 1:

1 $$ {C}_{t1}=\alpha +{\beta}_1I+{\beta}_2{C}_{t0}+\varepsilon $$

Where I is the group assignment and C indicates costs in the period after (t1) or before (t0) randomization.

For sensitivity analyses, we expanded the regression analysis with other covariates, such as level of education, Charlson Comorbidity Index, and type of homelessness as shown in model 2:

2 $$ {C}_{t1}=\alpha +{\beta}_1I+{\beta}_2{C}_{t0}+{\beta}_3X+\varepsilon $$

Where X is a vector of covariates.

In addition, health care costs were divided into the three categories (acute, elective, and social) and compared in a linear regression model. HRQoL values were used for computation of QALYs using Danish preference weights [36, 37]. Incremental cost-effectiveness ratios (ICERs) were computed by subtracting the QALY gain in controls from the QALY gain in cases, obtaining the incremental effectiveness; the incremental costs were achieved in a similar manner. Finally, the incremental QALY gain was divided by the incremental costs [35].

The ICER is a fraction; therefore, it is not straightforward to obtain confidence limits for the estimate. Therefore, the costs, effects, and ICERs were bootstrapped, drawing 10,000 samples with replacement, and the resulting ICERs plotted in a cost-effectiveness plane. In this exercise, whether the intervention is cost-effective is displayed visually, and the bootstrapping results in confidence interval values for the costs, effects, and ICERs [38]. Bootstrapping was conducted on both the complete case data set (*N* = 40) and a data set with imputed QALY values in case of missing values (*N* = 89).

For statistical analyses, we used SAS® Enterprise Guide version 7.1 and SAS® 9.4. The bootstrapping of ICERs was conducted in STATA® MP 15.

## Results

A total of 96 homeless people (53 intervention, 43 control) from the Capital Region of Denmark were included in the study from April 2014 until follow-up ended in March 2016. The first six patients were all consecutively assigned to the intervention group, which explains the imbalance between the intervention and control group. The intervention group had a median length of stay of 12 (IQR 2–14) days. Four participants died during the follow-up period, two from each group. The data collected by social nurses in September 2015 gave information about 286 contacts with 260 individuals. The majority did not fulfill the inclusion criteria, as they were not homeless (66%), not mentally able (9%), or not physically able (5%). The remaining 20% were excluded because they left against medical advice, were transferred to another unit/hospital, required care 24 h a day, had language barriers, or were illegal immigrants.

### Participants

The 96 participants were similar in demographics and characteristics at baseline (Table 2) and were registered in 23 municipalities across Denmark, 1 came from Greenland, and 23 had an unknown municipality of residence, including 20 immigrants without a Danish social security number. We received data about municipal health care costs from nine municipalities equivalent to data from 52 individuals. The mean age of all participants was 48 years (SD = 10), 87 were men (91%), and they were primarily homeless (57%). Seventy percent of participants had a problematic use of alcohol, whereas only 13% had no problematic drug or alcohol use. The control group had more admissions 2 years prior to admission, mostly in psychiatric wards, than the intervention group (Table 2).

**Table 2:** Baseline demographic and clinical characteristics by group

Characteristics	Total *N* = 96	Intervention group (*n* = 53)	Control group (*n* = 43)
Age, mean (SD)	48 (10)	48 (10)	47 (10)
Men, n (%)	87 (91)	49 (92)	38 (88)
Had social security number, n (%)	76 (79)	41 (77)	35 (81)
Drug and alcohol use, n (%) (*n* = 74)^a^			
Alcohol	52 (70)	29 (67)	23 (74)
Benzodiazepines and/or hash	18 (19)	11 (21)	7 (16)
Central nervous system medication and/or opioid	17 (22)	10 (19)	7 (16)
No abuse	12 (13)	7 (13)	5 (12)
Housing status, n (%)			
Homeless	55 (57)	33 (62)	22 (51)
Functional homeless	13 (14)	8 (15)	5 (12)
Other^b^	12 (13)	5 (9)	7 (16)
Education, n (%)			
9 years or less	34 (35)	16 (30)	18 (42)
More than 9 years	21 (22)	14 (27)	7 (16)
Not listed/Unknown	41 (43)	23 (43)	18 (42)
Social benefits 52 weeks prior to baseline, n (%)			
Not eligible	23 (24)	14 (26)	9 (21)
Eligible	73 (76)	39 (74)	34 (79)
Cash assistance^c^			
No cash assistance	31 (42)	15 (38)	16 (47)
Cash assistance 52 weeks	23 (32)	14 (36)	9 (26)
Cash assistance between 1 and 51 weeks	19 (26)	10 (26)	9 (26)
Disability pension^c^			
No disability pension	56 (77)	31 (79)	25 (74)
Disability pension more than 1 week	17 (23)	8 (21)	9 (26)
Other benefits^c^	8 (11)	4 (10)	4 (12)
Hospital admissions 2 years prior to inclusion, n (%)			
Somatic			
1–3 admissions	32 (33)	18 (34)	14 (33)
> 3 admissions	41 (43)	22 (42)	19 (44)
Psychiatric			
1–3 admissions	13 (14)	8 (15)	5 (12)
> 3 admissions	16 (17)	7 (13)	9 (21)
Psychiatric diagnosis, n (%)	46 (48)	24 (44)	22 (51)
Charlson Comorbidity Index^d^	0,74	0,63	0,86

^a^Information on drug and alcohol use based on 74 of the 96 participants

^b^Not registered in the Danish Population Registry, but do not see themselves as homeless

^c^Percentages of cash assistance, disability pension, and other benefits were calculated from the 73 persons eligible for social benefits

^d^Charlson Comorbidity Index score and psychiatric diagnosis was estimated using data from 2009 until inclusion

### Health care costs

The economic evaluation of costs for 3 months showed that the control group had on average a €4761 (*p* = 0.10) higher cost than the intervention group (Table 3). The cost difference was significant when looking at costs for 6 months, when the cost difference was €8515 (*p* = 0.04), and after 12 months, when the control group had €12,603 (*p* = 0.03) higher costs on average. In the sensitivity analysis, in which we adjusted for covariates, the control group still had higher costs than the intervention group, €4328 (*p* = 0.13) after 3 months, €8161 (*p* = 0.05) after 6 months, and €10,687 (*p* = 0.06) after 12 months. Analyses with and without data from the Danish National Prescription Registry showed no differences.

**Table 3:** Cost computation regression models comparing control and intervention groups (average per person)

	Model 1 3 months €	Model 1 6 months €	Model 1 12 months €	Model 2 3 months €	Model 2 6 months €	Model 2 12 months €
Constant	1721	−603	− 2865	131	− 3086	− 4806
Intervention group	−4761	−8515*	−12,603*	−4328	−8161	−10,687
Costs before	0.15	0.25*	0.38*	0.08	0.17	0.25*
Education	–	–	–	1318	3266	4071
Charlson Comorbidity Index	–	–	–	1526*	2177*	5215*
Functionally homeless	–	–	–	9139*	7826	4912
Homeless other	–	–	–	256	− 1297	5464
Homeless missing	–	–	–	− 2787	− 4822	− 4867
*R*^2^	0.0575	0.1156	0.2038	0.1698	0.2055	0.3259

*p < 0.05

Model 1 is a difference in difference analysis and model 2 is difference in difference adjusted for level of education, Charlson Comorbidity Index and type of homelessness

### Health-related quality of life and ICER

The EQ-5D-5 L questionnaire were answered by 91 participants (95%) at baseline, 62 participants (65%) after 2 weeks, and 43 participants (61%) after 3 months. Among the 71 participants who were offered all three questionnaires, 38 (54%) answered all three. Both groups achieved minor QALY gains, which were slightly higher in the intervention group but not significant (Table 4).

**Table 4:** QALY gains and ICER (average per person)

	Model 1 3 months	Model 1 6 months	Model 1 12 months	Model 2 3 months	Model 2 6 months	Model 2 12 months
QALYs gained in intervention group	0.0016	0.0032	0.0063	0.0016	0.0032	0.0063
QALYs gained in control group	0.0007	0.0014	0.0027	0.0007	0.0014	0.0027
ICER	Dominant	Dominant	Dominant	Dominant	Dominant	Dominant

Model 1 is a difference in difference analysis and model 2 is difference in difference adjusted for level of education, Charlson Comorbidity Index and type of homelessness

The ICER was negative in all models and for all time horizons. A negative ICER is the result of either the intervention being better and cheaper, i.e. dominant, or vice versa [35]. In this case, the intervention is dominant, or more effective and cost-saving. In the underlying data, missing QALY values were imputed such that the data set used for bootstrapping contained 89 individuals. Though the bootstrapping did not render significant results, 68% of the bootstrap replications resulted in the ICER being dominant, and 96% of replications indicated that the intervention was cost saving. The complete case analysis showed similar results. The plot is shown in the supplementary information (Figure S1).

Table 5 shows how the costs were distributed among the categorized health services without being adjusted for healthcare use preceding inclusion in the study. The main difference was in the costs of acute admissions during the first 3 months, as the control group on average used €7139 per person, compared to €3048 in the intervention group. Costs for targeted services in the municipalities were more than twice as high for the control group (€3573) as the intervention group (€1605). Costs for in-hospital days after inclusion were related to patients waiting for an “alternative level of care” and were €814 for the control group and €255 for the intervention group. Rehabilitation, drug and alcohol therapy, and general care service expenditures were higher in the intervention group. The supplementary material (Table S1) shows the services provided at the Medical respite center.

**Table 5:** Elective, acute, and social costs at 3 and 6 months (€, crude average per person)

	3 months			6 months		
	Intervention €	Control €	*P*-value	Intervention €	Control €	*P*-value
Elective health costs						
General practitioner	40	36	0.849	92	62	0.3943
Outpatient visits^a^	623	708	0.794	798	1135	0.4079
Elective hospitalization	419	768	0.514	568	909	0.545
Rehabilitation	–	–	–	1047	0	0.2945
Alcohol/drug abuse treatment	–	–	–	898	177	.3888
Acute health care costs						
Acute admission	3048	7139	0.0746	4819	12,158	0.0487
Emergency department visit	164	256	0.356	307	431	0.447
In-hospital days after inclusion	255	814	0.0901	–	–	–
Social costs						
General care services	938	150	0.3429	1047	153	0.2945
Targeted care services	1605	3573	0.1598	2879	5328	0.2137
Medical respite care	1260	0	–	1260	0	–
Delivery of prescription drugs	96	109	0.82	164	183	0.82
Crude total	8448	13,553	0,07	13,045	20,536	0,1

^a^Somatic and psychiatric out-patient visit

## Discussion

“Bridge Copenhagen – medical respite care for homeless people” is the first randomized study to investigate the cost-effectiveness of a post-hospital medical respite care stay for homeless people. After 3 months, the costs in the intervention group were on average €4761 lower per person than the costs in the control group, but not significant. However, when we expanded the analysis time frame to 6 and 12 months, we found that the difference in costs between the groups steadily increased and were significant after both 6 and 12 months. When adjusting for length of education, the Charlson Comorbidity Index score, and homeless situation, the control group still had higher costs, but the difference was significant only at 6 months.

Both groups had a small, but not significant, gain in QALYs. There was a difference between the two groups in QALYs, which could be explained by the difference in the Charlson Comorbidity Index at baseline; the control group had worse health status at randomization. The cost-effectiveness analysis showed that the intervention was dominant for all models and time horizons. The cost difference was significant at 6 and 12 months, whereas the QALY difference was not significant, rendering the ICER as not significant. However, the consistent result that a post-hospital medical respite care stay is less costly while not inferior to usual care indicates the cost-effectiveness of the intervention.

There was a tendency that the intervention group had fewer costs related to acute admissions and targeted services in the municipalities and had fewer in-hospital days after inclusion. This could be due to health care services offered at the post-hospital medical respite care center enhancing the possibility of full recovery. At the same time, the intervention group had higher costs for general care services, rehabilitation, and treatment of substance abuse problems. One of the key results of the qualitative study by Pedersen et al., who studied the same respite care center, was that a post-hospital medical respite care stay allowed time for reflection on, for example, addiction problems [17]. It appears that some of the homeless people attended drug or alcohol therapy after the respite care stay. Taken together, it seems that a post-hospital medical respite care stay can reduce costs and acute admissions and increase the number of persons receiving drug and alcohol abuse treatment.

The reduced use of health care services after a respite care stay is supported by previous studies [39–41]. Kertesz et al. found that being discharged to a homeless respite care program reduced the odds of being readmitted 50% following 3 months of inclusion, but the intervention was not cost-effective compared to discharging the homeless people to the streets and shelters. However, the intervention described in that study provided 24-h a day care with highly specialized interdisciplinary teams, allowing for more complex patient care [39]. Buchanon et al. reported that respite care for homeless people reduced costs because it reduced inpatient days [40]. Notably, the costs for medical respite care in Buchanan et al.’s study were half that of our intervention, and the cost for a day in the hospital was roughly estimated as being 3-times as expensive as in the present study. Furthermore, the average length of the respite care stay was 42 days [40]. Our eligibility criteria and the fact that most participants used alcohol may reflect that the participants in our study were a select group that demanded less intensive care. These important differences give rise to further considerations about which services should be provided at a post-hospital medical respite care center and when the marginal effect of one additional day approaches zero. In comparison with other interventions such as housing first we see similar results regarding hospital use [21], which underline the importance of temporary or stable housing as our study reports that 23% of the intervention group obtained temporary housing in continuation of the medical respite care stay.

This study has several limitations. First, it is unclear whether it is realistic to expect any changes in HRQoL in such a short time period as 3 months. Second, our results should be viewed as conservative estimates because Copenhagen offers many targeted services for homeless people, which together improve the chances of full recovery regardless of post-hospital medical respite care. Third, we did not evaluate whether the intervention entailed some patients receiving treatment faster because our target group could be discharged faster to an alternative level of care. Therefore, the full effect of medical respite care could be even more pervasive. Lastly, data from immigrants could be underreported because they do not occur in Danish registers. As the control group had a higher percentage of immigrants, the missing data may have resulted in an underestimation of the costs in the control group. We had no information on the length of homelessness prior to the medical respite care stay. In future studies with more participants, it would be relevant to include information on length of homelessness in the analysis. Lastly, the municipal costs are based on imprecise estimates and the accounting method varied between municipalities. However, because of the randomization, we expect that the lack of precision of these estimates impacts both groups similarly and, therefore, should not influence the economic evaluation.

Our study has several strengths. First, it was performed as a randomized controlled trial, which is the gold standard in medical research for investigating the effect of an intervention but a rarely used design to evaluate interventions for homeless people. Second, it was performed in a “real-world” setting, where the intervention was delivered by an NGO, rendering it generalizable and can be transferred to many parts of the world, because the results do not rely on a welfare system like the one we have in Denmark. Third, using Danish registries, it is possible to estimate differences in health care costs across different sectors and services. This allowed us to investigate whether a post-hospital medical respite care stay is cost-effective at a societal level. Lastly, the response rate for HRQoL was surprisingly high in this population compared to other studies; Hewet et al. were able to get in contact with approximately 25% of the homeless people for a 6-week follow-up [22]. The participants we failed to contact were primarily from the intervention group, which means our effect results could be underestimated. It is possible that the use of health care services is reflected in the HRQoL; therefore, future studies could expand the follow-up time to investigate whether HRQoL increases as the health care costs decrease. This study demonstrated that it is possible to perform a pragmatic randomized controlled trial with a low attrition rate in this socially stigmatized population, even though it is not a tradition in the social area. This leads to new opportunities for creating evidence-based interventions in an area that is driven mainly by experience.

## Conclusions

This study shows that post-hospital medical respite care for homeless people in a Danish setting leads to significant improvements in cost-effectiveness after 6 and 12 months. The differences in health care costs after 3 months follow-up also suggested cost-effectiveness, bud did not reach statistical significance. HRQoL showed small improvements in both groups and were highest in the intervention group, though not significant.

This study informs policy makers and health professionals who work with homeless people and strongly suggests that post-hospital medical respite care should be implemented in similar health care settings.

Future studies need to address the heterogeneity of the homeless population by either including a larger population or focusing on certain characteristics. Furthermore, future studies should investigate the potential benefit if street nurses and doctors can refer homeless people directly from the street to medical respite care, and thereby preventing hospital admissions.

## Data Availability

The data that support the findings of this study are available from Statistics Denmark, but restrictions apply to the availability of these data, which were used under license for the current study, and therefore are not publicly available. However, data are available from the authors upon reasonable request and with permission of Statistics Denmark.

## Abbreviations

CUA: Cost-utility analysis
HRQoL: Health-related quality of life
ICER: Incremental cost-effectiveness ratio
NGO: Non-governmental organization
QALY: Quality adjusted life years
RN: Registered nurse
